# Patient Survival With Extended Home Hemodialysis Compared to In-Center Conventional Hemodialysis

**DOI:** 10.1016/j.ekir.2023.09.007

**Published:** 2023-09-15

**Authors:** Ercan Ok, Cenk Demirci, Gulay Asci, Kivanc Yuksel, Fatih Kircelli, Serkan Kubilay Koc, Sinan Erten, Erkan Mahsereci, Ali Rıza Odabas, Stefano Stuard, Franklin W. Maddux, Jochen G. Raimann, Peter Kotanko, Peter G. Kerr, Christopher T. Chan, The Turkish Home Hemodialysis Initiative, Fatma Toz, Huseyin Toz, Mehmet Ozkahya, Meltem Sezis, Mumtaz Yilmaz, Mehmet Sukru Sever, Alaattin Yıldız, Sıddig Momin Adam, Mine Besler, Handan Ogunc, Mujdat Batur Canoz, Mustafa Eren, Melih Anil, Kezban Pinar Yeniay, Ismail Ozer, Pınar Ergin, Elif Arı Bakır, Habib Emre, Hüseyin Atalay, Cemal Kurt, Fatma Adam, Pinar Seymen, Numan Görgülü, Bahtisen Guven, Mustafa Keleş

**Affiliations:** 1 Ege University, Izmir, Turkey; 2 Fresenius Medical Care, Izmir, Turkey; 3 Fresenius Medical Care, Bad Homburg, Germany; 4 Fresenius Medical Care, Istanbul, Turkey; 5 Fresenius Medical Care, Gaziantep, Turkey; 6 Medeniyet University, Istanbul, Turkey; 7 Fresenius Medical Care, Boston, Massachusetts, USA; 8 Renal Research Institute, New York, NewYork, USA; 9 Monash University, Melbourne, Australia; 10 University of Toronto, Toronto, Ontario, Canada

**Keywords:** hemodialysis, home hemodialysis, hospitalization, matched case-control study, medication requirement, survival

## Abstract

**Introduction:**

More frequent and/or longer hemodialysis (HD) has been associated with improvements in numerous clinical outcomes in patients on dialysis. Home HD (HHD), which allows more frequent and/or longer dialysis with lower cost and flexibility in treatment planning, is not widely used worldwide. Although, retrospective studies have indicated better survival with HHD, this issue remains controversial. In this multicenter study, we compared thrice-weekly extended HHD with in-center conventional HD (ICHD) in a large patient population with a long-term follow-up.

**Methods:**

We matched 349 patients starting HHD between 2010 and 2014 with 1047 concurrent patients on ICHD by using propensity scores. Patients were followed-up with from their respective baseline until September 30, 2018. The primary outcome was overall survival. Secondary outcomes were technique survival; hospitalization; and changes in clinical, laboratory, and medication parameters.

**Results:**

The mean duration of dialysis session was 418 ± 54 minutes in HHD and 242 ± 10 minutes in patients on ICHD. All-cause mortality rate was 3.76 and 6.27 per 100 patient-years in the HHD and the ICHD groups, respectively. In the intention-to-treat analysis, HHD was associated with a 40% lower risk for all-cause mortality than ICHD (hazard ratio [HR] = 0.60; 95% confidence interval [CI] 0.45 to 0.80; *P* < 0.001). In HHD, the 5-year technical survival was 86.5%. HHD treatment provided better phosphate and blood pressure (BP) control, improvements in nutrition and inflammation, and reduction in hospitalization days and medication requirement.

**Conclusion:**

These results indicate that extended HHD is associated with higher survival and better outcomes compared to ICHD.

## Graphical abstract

**Figure:**
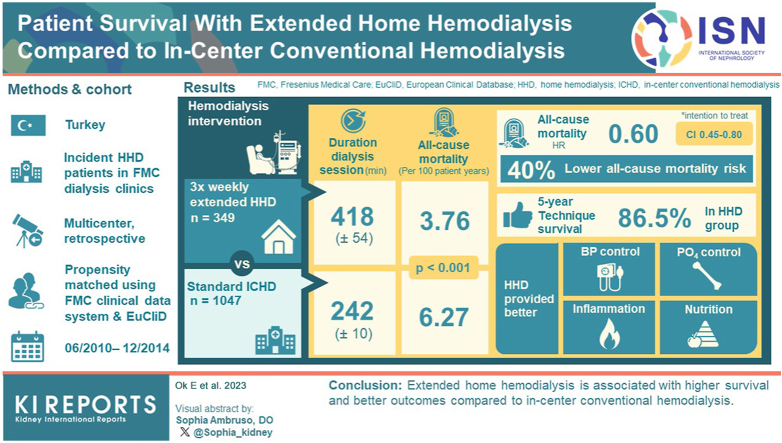

See Commentary on Page 2501

HD is a life-sustaining treatment, utilized by two-thirds of patients with end-stage kidney disease (ESKD). However, the typical regimen of thrice-weekly 3-hour to 4-hour ICHD is associated with high mortality, low quality of life, and high total cost.

Many attempts have been made to improve survival; however, most have not resulted in substantial improvement. Intensive dialysis regimens have become the focus of attention in recent years due to the growing body of evidence showing that more frequent and/or longer HD sessions are associated with improvements in a wide spectrum of outcomes.^1^, ^2^, ^3^, ^4^, ^5^, ^6^, ^7^, ^8^, ^9^, ^10^, ^11^

Home is the optimal place to receive more frequent and/or longer HD. It is more economical, comfortable and provides more successful patient compliance. HHD is a well-established method that has been used since the start of HD, although its prevalence has largely fluctuated over time. Following a sharp decline since the 1970’s, the popularity of HHD has increased again in recent years, as a result of both poor outcomes with ICHD and growing request for more cost-effective treatments.^12^ Importantly, the introduction of user-friendly and portable HHD machines has contributed greatly to the expansion of HHD programs.

Despite HHD being more economical than ICHD, utilization of HHD in low- and middle-income countries remains rare.^13^, ^14^, ^15^ The HHD program initiated in June 2010, as the first effort of its kind in Turkey, has shown a remarkable development over time, reaching 1218 patients as of December 31, 2021. To our knowledge, the HHD program recently implemented in Turkey may be the largest one among those in low- and middle-income countries.

HHD, more frequent and/or longer has been demonstrated to provide better BP and serum phosphate control, improvements in left ventricular hypertrophy and quality of life, as well as lower hospitalization and cost.^1^^,^^2^^,^^14^^,^^16^, ^17^, ^18^ Although retrospective studies have suggested that HHD improves patient survival, many of them had methodological shortcomings such as small sample size, short follow-up, residual confounding, or selection bias.

In this study, we investigated overall survival in patients on HHD compared to ICHD. We also explored technique survival, hospitalization, medication use, and changes in clinical and laboratory parameters.

## Methods

This is a retrospective, propensity score-matched cohort study comparing overall survival among patients receiving HHD and ICHD. All ethical and regulatory rules concerning patient data safety and privacy were met locally and all patients signed the appropriate informed consent form authorizing use of their clinical data for anonymized scientific research. The study was conducted according to the principles of the Declaration of Helsinki.

### Patient Population

All patients, who initiated HHD (patients on incident HHD) between June 2010 and December 2014 in Fresenius Medical Care dialysis clinics (*n* = 46) in Turkey were screened for the study (*n* = 362). All other patients on ICHD that were actively being treated in these clinics during the same time period were considered for the control group.

Inclusion criteria were age over 18 years and on ICHD for more than 3 months at the study initiation. Exclusion criteria were an HD frequency other than thrice-weekly, nurse-assisted HHD, and missing baseline data for the matching procedure.

**Figure 1:**
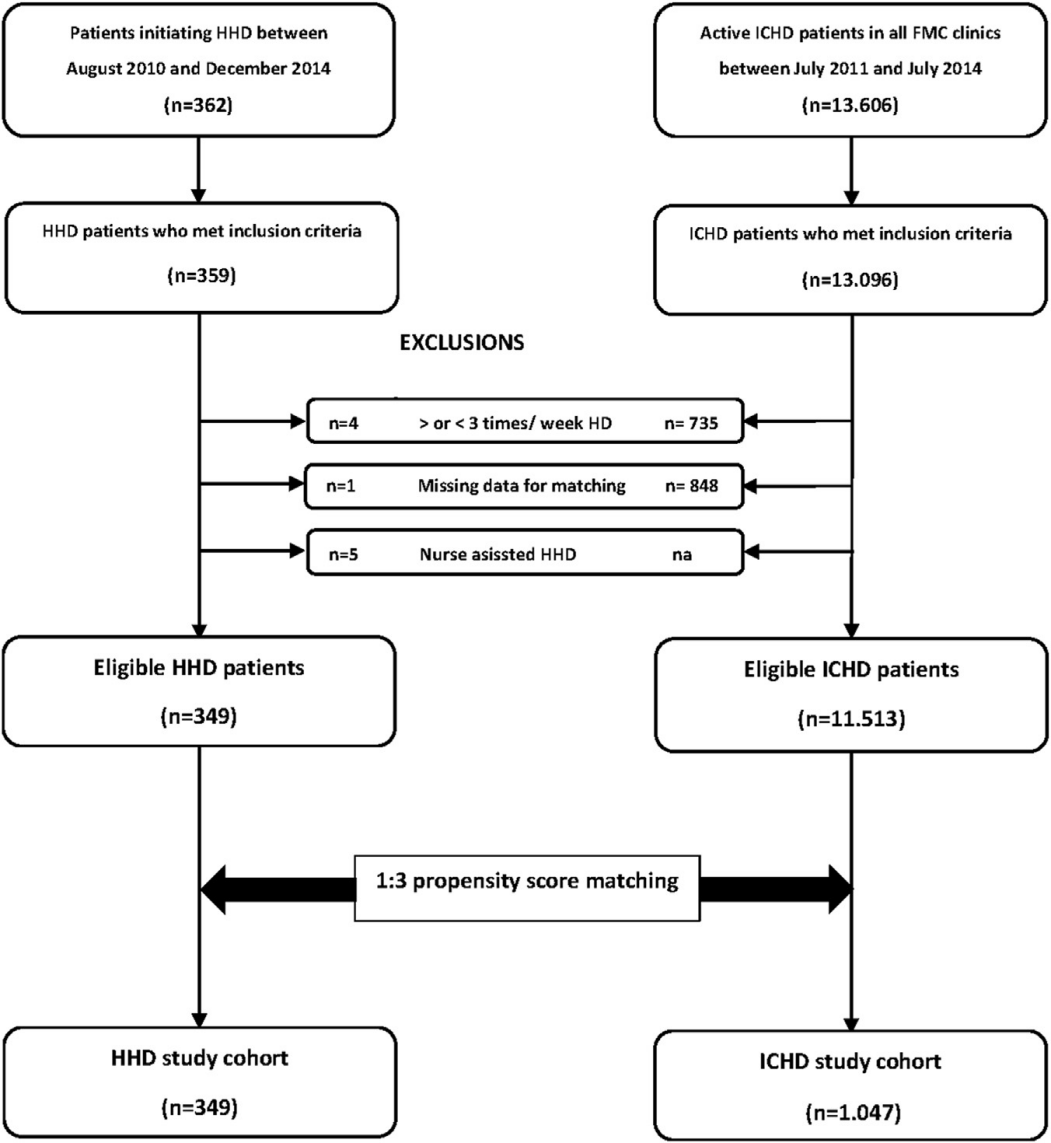
Flow chart of the study. FMC, Fresenius Medical Care; HD, hemodialysis; HHD, home hemodialysis; ICHD, in-center conventional HD.

A total of 349 patients on HHD who met eligibility criteria were enrolled in the study (Figure 1). The study entry date was defined by the starting date of the HHD treatment. HHD cases and ICHD controls were matched by initiation date and the propensity scores. Detailed information is given in the flow chart of the study (Figure 1 and Supplementary Figures S1–S4). The final study cohorts consisted of 349 patients on HHD and 1047 patients on ICHD. All patients were followed-up with until September 30, 2018.

### Study Design

All data were obtained from the Fresenius Medical Care clinical data system, European Clinical Database, which is validated for epidemiological studies.^19^

Demographic, clinical, biochemical, or dialysis treatment parameters and medication usage were collected at the baseline, monthly during the first 3 months, then quarterly until the 24th month and then twice a year. In patients on HHD, baseline data collection was performed within 3 months before initiation of dialysis at home, while the patient is still on ICHD; the data closest to initiation of HHD was used.

Baseline comorbidities, including myocardial infarction, congestive heart failure, cerebrovascular disease, peripheral vascular disease, chronic obstructive pulmonary disease, and malignancy were also collected. Patients who had at least 1 of the following: myocardial infarction, congestive heart failure, cerebrovascular disease, or peripheric artery disease were considered as having cardiovascular disease at baseline.

Ultrapure dialysate, the same HD machines (Fresenius 4008S and 5008S) and dialyzer types (high-flux polysulfone membrane) were used in both groups. Dialysate flow rates were 300 and 500 ml/min in the HHD and the ICHD group, respectively. Overall, physicians were asked to follow Kidney Disease Outcomes Quality Initiative and/or Kidney Disease: Improving Global Outcomes Guidelines. All blood samples were analyzed centrally by SYNLAB, Turkey, registered to external quality control programs.

### Study Outcomes

The primary outcome was all-cause mortality. All deaths observed during follow-up (up to 8 years) were recorded. Secondary outcomes were technique survival, composite of death and technique survival, hospitalization, and changes in clinical/laboratory/dialysis treatment parameters and required medications.

### Matching Procedures

#### Matching for the Primary Outcome

Age, gender (male and female), diabetes, ESKD duration, body mass index (BMI), systolic BP, albumin, and phosphate were significantly different between the HHD and in the ICHD groups before matching (standardized difference >10%); hemoglobin (Hb), serum C-reactive protein (CRP), and vascular access were comparable (Supplementary Table S1). Propensity score matching procedure was used to select 1047 patients on ICHD from the pool to create a comparable cohort to 349 patients on HHD. Covariates used for this purpose were age, gender, diabetes, BMI, ESKD duration, albumin, phosphate, systolic BP, and vascular access. We estimated the propensity score at the start of follow-up with a logistic regression model that included all listed covariates as predictors. Although the 2 groups were similar regarding vascular access, it was included in the matching procedure because of its well-known role as an independent risk factor for mortality.^20^^,^^21^ Comorbidities were not used in the matching process, because we suspected some deficiencies in the database regarding comorbidities of cases screened for inclusion. However, after matching, the comorbidity data of all selected patients on HHD and patients on ICHD (*n* = 1396) were individually screened in the written records and completed to allow for accurate characterization of the cohort. We matched patients on ICHD and patients on HHD using the estimated propensity score utilizing the Greedy Nearest Neighbor Matching algorithm in a 3:1 ratio for patients on ICHD and patients on HHD (SAS 9.4 software, SAS Institute Inc, Cary, NC).^22^ The appropriateness of the matching performance was evaluated using standardized differences. A difference of less than 10% was considered successfully matched.

#### Matching to Evaluate the Changes in Clinical and Laboratory Parameters

There were 252 patients on HHD and 768 patients on ICHD who completed 12 months of follow-up period and who had clinical and laboratory results at the 12th month. In order to compare the changes in clinical/biochemical parameters between the 2 groups, the new propensity score matching procedure was employed to select patients on ICHD (*n* = 756) matched with patients on HHD (1:3) by using same covariates utilized for the assessment of primary outcome.

### Statistical Analyses

Descriptive statistics are reported as mean (SD) for normally distributed variables and median (interquartile range) for the variables not normally distributed; categorical variables were reported as percentages (%). A *P-*value ≤ 0.05 was considered statistically significant. Between and within groups comparisons were performed using independent and dependent sample t-tests, Mann Whitney U test for continuous variables, and chi-square and McNemar tests for categorical variables.

The primary analysis was performed by using the intention-to-treat principle. We followed all patients on HHD and their matched patients on ICHD from the initiation of HHD in index HHD cases until death, kidney transplantation, loss to follow-up, or September 30, 2018.

As-treated survival analyses were performed with the censoring the data for mortality analysis at the time of death, change of dialytic modality (from HHD to ICHD or peritoneal dialysis, from ICHD to HHD or peritoneal dialysis), kidney transplantation, loss to follow-up or September 30, 2018. Deaths that occurred within 3 months after change of dialytic modality were not assigned to the first HD modality.

Survival analyses were performed using the Kaplan-Meier method. Cox proportional hazard models were utilized to assess overall survival in propensity score matched patients. After checking proportional hazard assumption, the results were interpreted using HR. In addition, the Cox proportional hazards were adjusted for comorbidities (myocardial infarction, congestive heart failure, cerebrovascular disease, peripheral vascular disease, and malignancy), smoking status, CRP and Hb, which were not used in propensity score matching procedure.

For technique survival analyses, follow-up was censored at the time of death and transplantation. In the composite patient and technique survival assessment, patients were followed-up with until the first occurrence of technique failure and/or death and were censored at kidney transplantation.

Hospitalization rate and hospitalization days per patient-year were compared using negative binomial analysis.

We performed prespecified subgroup analyses according to median age, gender, ESKD duration, presence of diabetes and cardiovascular disease (CVD), BMI, and vascular access according to the intent-to treat analyses. Fully adjusted results were reported. Differences between the subgroups were checked by interaction analyses by entering product terms into Cox regression models.

Analysis of covariance models were used to assess the between-treatment group differences in the changes from baseline to the 12th month in the clinical/biochemical parameters in patients who completed 12 months of follow-up. Analysis of covariance models for normally distributed data, comparisons of mean changes from baseline to the 12th month between groups were adjusted with baseline values. Median values were used for nonnormally distributed variables. For the mean difference in change of these nonnormally distributed variables between groups, we used change in geometric means of log-transformed data as percentages in analysis of covariance models. SPSS Statistics 25.0 (SPSS Inc, Chicago, IL) was used for all analyses except the matching procedure.

## Results

Baseline characteristics were similar between the HHD and ICHD groups after matching procedure (standardized differences <5%) (Table 1). At baseline, nearly the entire population had been treated with 3 to 5 hours of ICHD 3 times a week.

**Table 1:** Baseline parameters of matched HHD and ICHD groups

Characteristics	Home HD (*n* = 349)	In-center HD (*n* = 1047)	*P-*value
Age (yrs)	43.7 (11.8)	44.2 (12.6)	0.46
Female (%)	33.0	33.1	0.97
Diabetes (%)	14.0	13.6	0.82
Duration of ESRD (mo)	58 (24, 118)	64 (23, 125)	0.43
BMI (kg/m^2^)	25.0 (5.2)	25.1 (5.6)	0.90
Smoking (%)			
Never	63.6	62.0	
Ex-smoker	18.1	21.0	0.46
Current smoker	18.3	17.0	
Primary cause of ESRD (%)			
Diabetes	9.1	10.4	0.49
Hypertension	19.4	22.5	0.22
Glomerulonephritis	13.5	10.8	0.16
Polycistic kidney disease	6.2	5.6	0.71
Obstructive nephropathy	3.8	3.4	0.71
Other	3.8	3.7	0.84
Unknown	44.2	43.6	0.87
HD treatment			
3-times weekly 3 to 5 hours HD (%)	98.6	99.3	0.18
Effective blood flow rate (ml/min)	352 (38)	351 (38)	0.58
Vascular Access			
Fistula (%)	87.4	88.0	0.92
Graft (%)	2.3	2.7	0.69
Catheter (%)	10.3	9.3	0.56
Clinical and laboratory values			
Post-HD weight (kg)	69.4 (16.9)	68.7 (17.1)	0.48
IDWG (kg)	2.35 (0.96)	2.25 (0.95)	0.09
IDWG (% of post-HD weight)	3.44 (1.28)	3.36 (1.38)	0.32
Pre-HD systolic BP (mm Hg)	130 (20)	130 (17)	0.91
Pre-HD urea (mg/dl)	123 (30)	125 (31)	0.53
Pre-HD creatinine (mg/dl)	8.90 (2.05)	8.99 (2.31)	0.48
Calcium (mg/dl)	8.94 (0.82)	8.89 (0.78)	0.32
Phosphate (mg/dl)	5.06 (1.40)	5.11 (1.45)	0.55
Parathyroid hormone (pg/ml)	384 (185, 661)	382 (210, 673)	0.20
Alkaline phosphatase (U/l)	96 (73, 139)	101 (65, 144)	0.32
Urea reduction ratio (%)	75.7 (7.2)	75.7 (7.2)	0.99
Equilibrated Kt/V	1.51 (0.35)	1.49 (0.31)	0.46
Albumin (g/dl)	4.11 (0.33)	4.11 (0.33)	0.82
Hb (g/dl)	11.5 (1.5)	11.5 (1.5)	0.97
Ferritin (ng/ml)	611 (346, 896)	604 (368, 920)	0.73
Transferrin saturation (%)	31.7 (16.4)	30.6 (16.1)	0.30
Iron (μg/dl)	67.6 (32.5)	67.4 (34.4)	0.95
Bicarbonate (mEq/l)	21.2 (2.7)	21.5 (2.6)	0.12
CRP (mg/dl)	0.32 (0.12, 0.87)	0.33 (0.09, 0.95)	0.93
Neutrophil to lymphocyte ratio (%)	2.37 (1.60, 3.23)	2.24 (1.61, 3.23)	0.41
Comorbidities			
Myocardial infarction (%)	12.3	11.8	0.78
Congestive heart failure (%)	9.2	10.0	0.66
Cerebrovascular disease (%)	6.3	5.9	0.80
Peripheric artery disease (%)	4.9	6.2	0.35
Cardiovascular disease (%)^a^	21.5	20.5	0.69
Malignancy (%)	1.7	1.8	0.90
COPD (%)	7.2	6.9	0.86

BMI, body mass index; COPD, chronic obstructive pulmonary disease; CRP, C-reactive protein; ESRD, end-stage renal disease; HD, hemodialysis; IDWG, interdialytic weight gain; SBP, systolic blood pressure.

Data are presented as mean (SD) or median (interquartile range) or percentage (%), as appropriate.

a Patients who have at least 1 of the followings: myocardial infarction, congestive heart failure, cerebrovascular disease or peripheric artery disease.

During the follow-up, the frequency of HD sessions remained thrice-weekly for the majority of the population, except for 14 patients on HHD (every other day or 4 times a week) (4.0%); these cases were not excluded in the analyses. Time-averaged session durations were 418 ± 54 minutes in patients on HHD and 242 ± 10 minutes in patients on ICHD (*P* < 0.001). In 86.8% of patients on HHD, session duration was over 6 hours, most of them on nocturnal HHD. Mean eKt/V values were higher in the HHD group. In the vast majority of patients on HHD, dialysis treatment was administered by the patients themselves and in 41 patients (11.7%) by family members.

Forty patients on HHD (11.5%) switched dialysis modality, 38 to ICHD and 2 to peritoneal dialysis. Thirteen patients on ICHD switched to HHD (1.2%). Five patients on ICHD were lost to follow-up. Transplantation rates were 5.3 and 5.5 per 100 patient-year in the HHD and in the ICHD groups, respectively.

### Overall Survival

In intention-to-treat analyses, median follow-up was 55.4 (34.2, 69.8) and 54.8 (28.7, 63.9) months for the HHD and the ICHD groups, respectively (*P* = 0.13).

**Figure 2:**
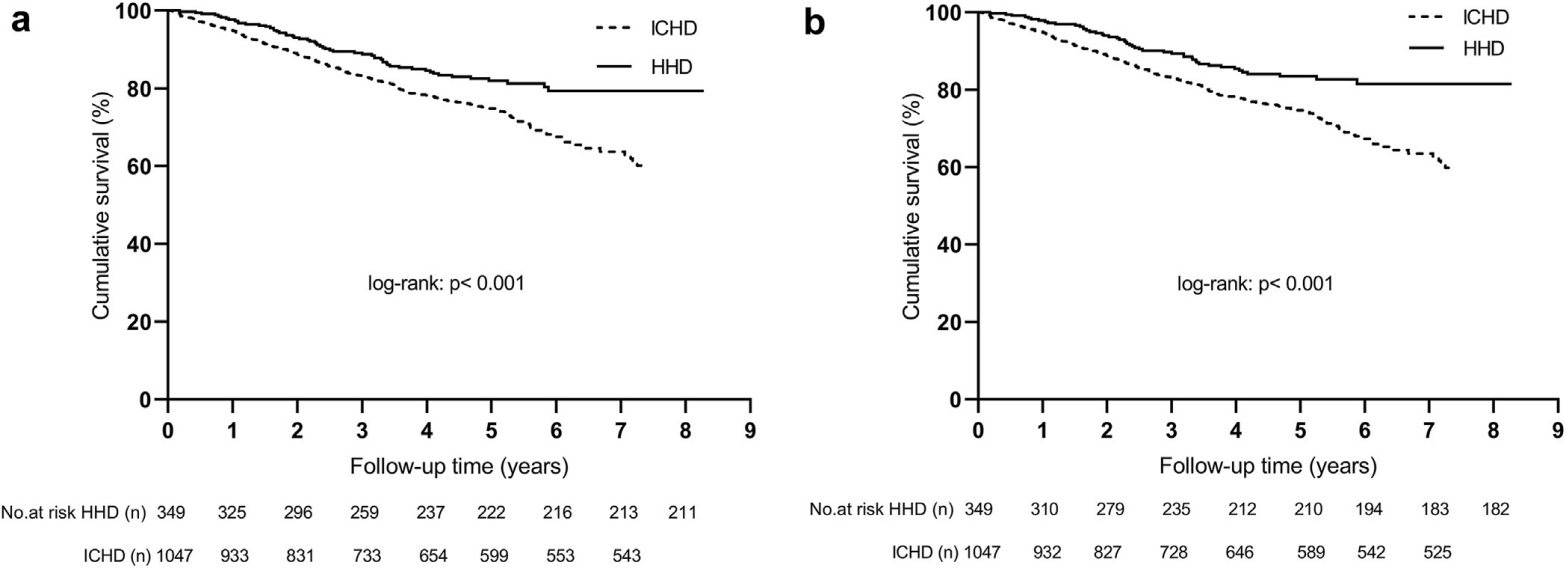
Kaplan Meier survival analyses in the HHD and the ICHD groups. (a) Intention-to-treat; and (b) as-treated. HHD, home hemodialysis; ICHD, in-center conventional HD.

All-cause mortality rate was 3.76 per 100 patient-years with HHD (*n* = 57) and 6.27 per 100 patient-years with ICHD (*n* = 269) (*P* < 0.001). At the end of follow-up, overall survival rates were 79.3% in the HHD arm and 59.1% in the ICHD arm (log-rank = 12.4, *P* < 0.001) (Figure 2a). In the HHD group, there were no deaths from a procedural adverse event (in over 210,000 HHD sessions).

All-cause mortality risk was lower by 40% in the HHD group compared to the ICHD group (HR = 0.60; 95% CI 0.45–0.80; *P* < 0.001).

Adjusting for variables not used in propensity score matching (comorbidities, smoking, CRP, and Hb) did not change the results (HR = 0.61; 95% CI 0.46–0.81; *P* = 0.001). Even when further corrections were made for all variables, the results remained similar (HR = 0.65; 95% CI 0.48–0.86; *P* = 0.003).

Age, vascular access, serum albumin, phosphate, CRP, diabetes, congestive heart failure, and malignancy were independent predictors of all-cause mortality in intention-to-treat analyses (model chi-squared: 250, *P* < 0.001).

In as-treated analyses, overall survival was 81.5% with HHD and 58.8% with ICHD (log-rank = 15.5, *P* < 0.001) (Figure 2b). HHD was associated with a 46% relative risk reduction in all-cause mortality (HR = 0.54; 95% CI 0.40–0.74; *P* < 0.001). Exclusion of deaths within the first 3 months of the study did not materially change the results (HR = 0.67; 95% CI, 0.50–0.90; *P* = 0.007).

All 13 patients on ICHD who switched to HHD treatment survived during follow-up. Among patients on HHD who switched to ICHD (*n* = 38), 9 patients died during follow-up; all deaths occurred after the first 3 months of modality change.

### Technique Survival

Within the follow-up period (48.2 ± 24.4 months), technical failure occurred in 40 patients on HHD (11.5%) due to logistical, medical, and social reasons (Supplementary Table S2). Patients who discontinued HHD had longer ESKD duration (101 ± 81 vs. 76 ± 69 months, *P* = 0.04) and higher CVD rates (40.0% vs. 19.1%, *P* = 0.01). There were no differences between the patients who continued HHD or switched to another modality regarding age, gender, presence of diabetes, and receiving family member assistance for dialysis.

**Figure 3:**
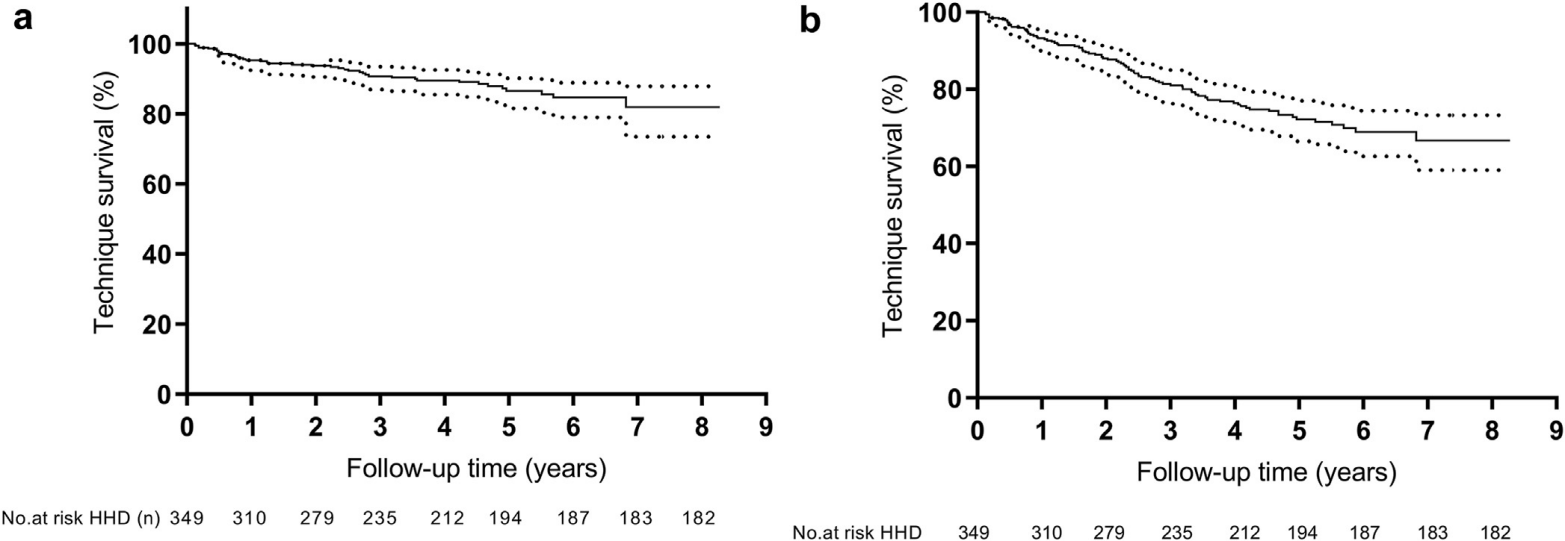
Technique survival analyses in the HHD group. (a) Death and transplantation-censored technique survival. (b) Composite technique survival. HD, hemodialysis; HHD, home hemodialysis.

Death and transplant-censored technique survival at 1, 3, 5, and 7 years of follow-up were 95.3, 90.7, 86.5, and 81.9%, respectively. Composite death and technique survival at 1, 3, 5, and 7 years during follow-up was 93.3, 81.1, 72.2, and 66.7%, respectively (Figure 3a and b).

### Hospitalization

Annual hospitalization rates were lower with HHD (0.31; 95% CI 0.23–0.40) compared to ICHD (0.43; 95% CI 0.36–0.51) (*P* = 0.03). Patients on HHD had an all-cause hospitalization rate of 2.2 hospital d/yr (95% CI 1.4–3.0) compared with 4.6 hospital d/yr (95% CI 3.6–5.6) of patients on ICHD (*P* < 0.001).

### Subgroup Analyses

**Figure 4:**
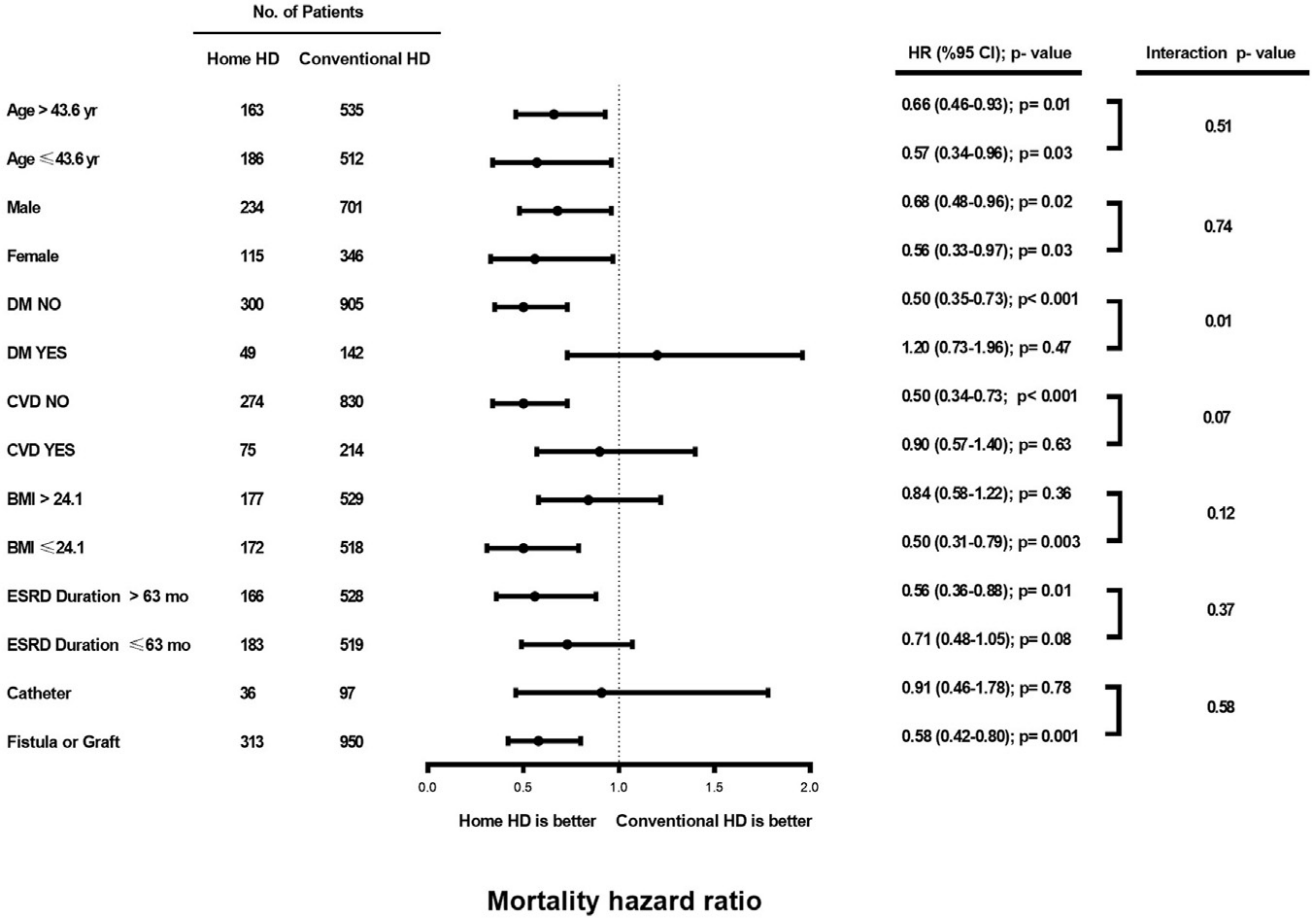
Forest plot (subgroup analyses). BMI, body mass index; ESRD, end-stage renal disease; HD, hemodialysis.

There was an interaction between the treatment group and the presence of diabetes. HHD treatment was associated with lower mortality risk in both genders and in all ages (Figure 4). The decrease of mortality risk with HHD was significant in patients with ESKD duration >63 months; trending toward lower mortality risk in patients with ESKD duration ≤63 months (*P* = 0.08). HHD treatment was associated with better survival in patients with BMI ≤24.1 kg/m^2^, but not in patients with BMI >24.1kg/m^2^. HHD was not related to higher survival in patients with diabetes, CVD, and catheter.

### Changes in Clinical and Laboratory Parameters in Patients who Completed 1-Year Follow-Up

At baseline, clinical and laboratory parameters were similar between groups; the differences in changes of these parameters within 12 months are presented in Table 2.

**Table 2:** Differences in the changes in laboratory and clinical parameters from baseline to 12th month between groups

Characteristics	Home HD (*n* = 252)	In-center HD (*n* = 756)	Mean difference between the groups (95% CI) (HHD vs. ICHD)	Adjusted mean difference between the groups^a^ (95% CI) (HHD vs. ICHD)	*P*-value
Anemia Control					
Hemoglobin (g/dl)					
Baseline	11.4 (1.5)	11.6 (1.5)			0.12
12th mo	11.3 (1.3)	11.6 (1.3)			<0.001
Change	−0.14 (1.40)	−0.04 (1.51)	−0.17 (−0.39 to 0.04)	−0.28 (−0.44 to −0.11)	0.001
Iron (μg/dl)					
Baseline	68.5 (31.5)	66.3 (33.3)			0.35
12th mo	67.1 (24.1)	71.0 (27.3)^b^			0.04
Change	−1.38 (33.6)	4.67 (33.8)	−6.05 (−10.9 to− 1.23)	−4.50 (−8.03 to −0.97)	0.01
Transferrin saturation (%)					
Baseline	31.9 (15.8)	30.1 (15.7)			0.12
12th mo	30.5 (11.5)	33.1 (13.6)^b^			0.003
Change	−1.40 (16.0)	2.94 (15.9)	−4.34 (−6.62 to −2.06)	−3.18 (−4.90 to −1.46)	<0.001
Ferritin (ng/ml)					
Baseline	626 (366, 904)	600 (367, 916)			0.80
12th mo	583 (384, 893)	657 (393, 931)			0.11
Change^c^, (%) (95% CI)	−8.07 (−5.88 to 0.48)	3.46 (−1.61 to 8.80)	−11.1 (−19.7 to −1.72)	−10.6 (−18.7 to −1.80)	0.01
ESA use (% of patients)					
Baseline	55.9	56.8			0.81
12th mo	37.1^b^	56.2			<0.001
IV iron use (% of patients)					
Baseline	41.6	45.9			0.23
12th mo	31.2^d^	45.5			<0.001
Mineral Metabolism					
Phosphate (mg/dl)					
Baseline	5.09 (1.35)	5.11 (1.46)			0.84
12th mo	4.46 (1.17)^b^	5.26 (1.26)^e^			<0.001
Change	−0.63 (1.45)	0.14 (1.38)	−0.77 (−0.97 to −0.57)	−0.79 (−0.94 to −0.63)	<0.001
Calcium (mg/dl)					
Baseline	8.94 (0.77)	8.92 (0.78)			0.69
12th mo	9.07 (0.70)^f^	8.98 (0.79)^d^			0.11
Change	0.13 (0.73)	0.06 (0.76)	0.07 (−0.04 to 0.18)	0.08 (−0.01 to 0.17)	0.09
PTH (pg/ml)					
Baseline	391 (213, 665)	392 (226, 666)			0.38
12th mo	348 (199, 591)	343 (215, 615)			0.52
Change^c^, (%) (95% CI)	−4.55 (−13.3 to 5.04)	−5.44 (−10.6 to 0.09)	0.93 (−9.81 to 13.0)	−2.74 (−2.15 to 7.68)	0.59
Alkaline phosphatase (U/l)					
Baseline	103 (74, 143)	100 (76, 143)			0.67
12th mo	117 (86, 169)^b^	105 (81, 147)^g^			0.008
Change^c^, (%) (95% CI)	17.5 (11.7 to 23.6)	4.81 (1.58 to 8.14)	12.1 (5.41 to 19.2)	10.9 (5.00 to 17.2)	<0.001
Phosphate binder use (% of patients)					
Baseline	74.4	78.0			0.24
12th mo	5.9^b^	76.4			<0.001
Vitamin D use (% of patients)					
Baseline	65.1	63.1			0.57
12th mo	44.3^b^	64.5			<0.001
Calcimimetic use (% of patients)					
Baseline	6.7	7.8			0.57
12th mo	1.7^h^	11.2^i^			<0.001
Nutrition And Inflammation Status					
Post-HD BW (kg)					
Baseline	69.6 (16.4)	69.2 (17.6)			0.75
12th mo	70.2 (16.8)^j^	69.3 (17.6)			0.48
Change	0.57 (3.00)	0.06 (3.34)	0.51 (0.04 to 0.98)	0.51 (0.05 to 0.98)	0.03
Albumin (g/dl)					
Baseline	4.10 (0.34)	4.12 (0.31)			0.42
12th mo	4.14 (0.30)^j^	4.12 (0.31)			0.22
Change	0.04 (0.30)	−0.004 (0.30)	0.05 (0.003 to 0.09)	0.04 (−0.0002 to 0.07)	0.05
Bicarbonate (mEq/l)					
Baseline	21.4 (2.66)	21.4 (2.53)			0.91
12th mo	21.7 (2.46)	20.4 (2.28)^b^			<0.001
Change	0.31 (3.18)	−1.03 (2.67)	1.34 (0.94 to 1.75)	1.33 (1.02 to 1.64)	<0.001
CRP (mg/dl)					
Baseline	0.31 (0.12, 0.79)	0.31 (0.10, 0.90)			0.86
12th mo	0.35 (0.13, 0.81)	0.31 (0.10, 1.04)			0.95
Change^c^, (%) (95% CI)	6.02 (−16.0 to 33.8)	4.21 (−9.5 to 19.9)	1.75 (−23.0 to 34.4)	1.23 (−18.3 to 25.4)	0.91
Neutrophil to lymphocyte ratio (%)					
Baseline	2.38 (1.59, 3.23)	2.24 (1.63, 3.17)			0.38
12th mo	2.19 (1.62, 2.83)	2.43 (1.80, 3.30)^b^			<0.001
Change^c^, (%) (95% CI)	−4.72 (−11.5 to 2.52)	13.4 (8.61 to 18.4)	−16.0 (−22.9 to −8.44)	−13.5 (−18.8 to −7.85)	<0.001
BP and volume status					
Systolic BP (mm Hg)					
Baseline	130.2 (18.6)	129.4 (16.3)			0.48
12th mo	128.5 (17.5)^k^	129.8 (15.8)			0.27
Change	−1.68 (13.2)	0.47 (11.6)	−2.15 (−3.80 to −0.50)	−1.92 (−3.44 to −0.40)	0.01
IDWG (% of post-HD BW)					
Baseline	3.41 (1.31)	3.34 (1.29)			0.48
12th mo	3.78 (1.34)^b^	3.39 (1.18)			<0.001
Change	0.38 (1.14)	0.04 (0.97)	0.33 (0.19 to 0.48)	0.36 (0.23 to 0.49)	<0.001
Ultrafiltration rate (ml/h/kg)					
Baseline	10.7 (3.15)	10.3 (3.42)			0.10
12th mo	6.27 (1.99)^b^	10.6 (3.18)^b^			<0.001
Change	−4.42 (2.49)	0.30 (2.33)	−4.73 (−5.07 to −4.39)	−4.58 (−4.88 to −4.29)	<0.001

BP, blood pressure; BW, body weight; CI, confidence interval; CRP, C-reactive protein; ESA, erythropoiesis-stimulating agents; HD, hemodialysis; HHD, home HD; ICHD, in-center HD; IDWG, interdialytic weight gainIV, intravenous; PTH, parathyroid hormone.

Data are presented as the mean (SD) or percentage (%) or median (interquartile range).

a Adjusted for the baseline level of the factor analyzed.

b *P* < 0.001.

c % changes in geometric means.

d *P* = 0.02.

e *P* = 0.004.

f *P* = 0.005.

g *P* = 0.003.

h *P* = 0.002.

i *P* = 0.001.

j *P* = 0.03.

k *P* = 0.04 within group.

At baseline, mean Hb levels tended to be lower in patients on HHD than in patients on ICHD. However, the proportion of patients with Hb level between 10 to 12 g/dl at 12 months was similar in the 2 groups (54.0% vs. 55.4%; *P* = 0.71). The utilization of erythropoiesis-stimulating agents decreased from 55.9% to 37.1% in patients on HHD; iron use was less than in patients on ICHD.

Mean phosphate levels declined in the HHD group and increased in the ICHD group. With HHD, the use of phosphate binder, vitamin D and calcimimetic decreased.

Post-dialysis body weight and albumin level increased in the HHD group. Adjusted difference was significant for post-dialysis body weight (*P* = 0.03), at borderline significance for albumin (*P* = 0.05). The difference between the changes of neutrophil/lymphocyte ratio in the 2 groups was significant.

Mean systolic BP decreased in the HHD group and did not change in the ICHD group. Changes in systolic BP between the groups were different.

## Discussion

In this study, we found that HHD was associated with a higher survival rate during follow-up of up to 8 years. Intention-to-treat analysis indicated a 40% reduction in the risk of all-cause mortality in patients on HHD compared to patients on ICHD.

None of the randomized trials on HHD were designed to have the power to investigate patient survival.^2^^,^^16^^,^^17^^,^^23^^,^^24^ A randomized trial on this topic seems unlikely given the previous recruitment difficulties.^25^ Several observational studies suggested a survival benefit with HHD.^26^, ^27^, ^28^, ^29^, ^30^ A recent review found a 13% to 52% reduction in mortality with HHD compared to ICHD in 10 of 13 included studies.^31^ Australian registry data suggest that both weekly session frequency greater than 3 and session length more than 4 hours may provide better patient survival.^30^ Both short daily and frequent nocturnal HHD have been reported to improve overall survival.^27^^,^^28^ Weinhandl *et al.* found that daily HHD is associated with a 13% lower risk for all-cause mortality than ICHD.^27^ Nesrallah *et al.*^28^ compared overall mortality in 338 frequent nocturnal patients on HHD (weekly session frequency 4.8 ± 1.1, session duration 7.4 ± 0.8 h) and 1.388 propensity score-matched patients on ICHD. In this well-designed study, the reduction in risk of death was 45% (95% CI 0.34–0.87), more pronounced than in our study (40%). This may be due to the lower session frequency of in our study.

Improvement of survival with HHD may be due to either performing HD at home and/or intensive HD (more frequent and/or extended). In the analysis of 262 patients who underwent short daily HD, Kjellstrand *et al.*^29^ demonstrated that performing HD at home and a longer weekly HD duration were predictors of lower mortality. In our study, it is not possible to distinguish the contributions of extended HD and performing HD at home on better survival with HHD because the session duration was prolonged in all patients on HHD and was within a narrow range of approximately 4 hours in all patients on ICHD. It can be speculated that patients on HHD may be living longer than patients on ICHD because of some unknown or unmeasured advantages of these patients themselves or some benefits arising from performing dialysis at home. However, given that similar successful results have been reported with in-center nocturnal HD, the duration of weekly HD session duration may be more important factor for the longer survival achieved with HHD.^3^^,^^4^

Our HHD population was younger and healthier compared to the general HD population, similar to the previous reports on patients on HHD.^2^^,^^26^, ^27^, ^28^^,^^32^ Propensity score matching enabled us to reduce selection bias by selecting carefully comparable individuals from a large pool of patients on ICHD. In order to eliminate the time effect, matching procedure was performed by the year of HHD initiation. Baseline characteristics of 2 groups were almost identical after matching (standardized differences <5%). Although comorbidities could not be used for matching, the frequency of comorbidities was found to be similar in the 2 groups after matching. Moreover, survival analyses were corrected with comorbidities. Patients on HHD were followed-up with from the beginning of HHD; the follow-up of each patient on ICHD began at the time when the index patient on HHD commenced HHD. We achieved a very low rate of lost to follow-up (0.36%). The capability of intent-to-treat analysis to distinguish the effects of HHD on survival was not limited because of the low discontinuation rate of HHD. Inclusion or exclusion of deaths in the first 3 months did not change the results. There was no difference in baseline characteristics of patients transplanted from the 2 groups (Supplementary Table S3). All the study centers were operated by the same large dialysis chain and had similar approaches to patient management. The homogeneity of HHD treatment is an advantage for assessing the effect of prolonged HHD. Finally, the comparable transplantation rates can be interpreted as an indicator that the groups are likely to be similar for other unmeasured prognostic variables. These strengths of this work make the results more reliable.

The survival benefit with HHD was present in several subgroups including both genders and age groups. As previously reported, the survival benefit with HHD was more pronounced in patients with longer ESKD duration.^28^ In this study, HHD did not provide a survival advantage in patients with a BMI >24.1 kg/m^2^. It can be estimated that longer follow-up is necessary to demonstrate the survival benefit with HHD in patients with higher BMI, which is protective for mortality among patients on dialysis.^33^ The number of cases was not sufficient to draw reliable conclusions about the effect of HHD in patients with diabetes, CVD history and catheter in this study.

Nine of 38 patients on HHD who returned to ICHD died during follow-up, whereas no patient was lost among those transferred from ICHD to HHD. Similarly, a recent study stated that mortality was significantly increased in patients returning from HHD to ICHD.^34^ Both an adverse health event leading to discontinuation of HHD and loss of benefit with HHD may contribute to this.

The 5-year technical survival rate of 86.5% in this cohort is in line with the Australia-New Zealand, UK, and Canada data,^35^, ^36^, ^37^ rather than the US data showing higher rates of HHD discontinuation.^38^ Diabetes, cardiac failure, older age, weekly session frequency greater than 3 and smoking/alcohol/drug use have been previously reported as the predictors of technique survival.^36^, ^37^, ^38^ We found longer ESKD duration and higher CVD frequency as predictors of HHD discontinuation.

Though a small randomized trial found no reduction in hospitalizations with frequent nocturnal HHD,^2^ a meta-analysis reported that nocturnal HHD was associated with fewer days of hospitalization per year, but no change in hospitalization rate.^18^ We found that HHD was associated with a 27.9% less hospitalization frequency and a 52.1% less hospital day compared to ICHD.

There are conflicting results about the effect of extended HHD on anemia.^39^^,^^40^ The results of the current study indicated lower Hb level in the HHD group, possibly due to lower use of both erythropoiesis-stimulating agents and iron. However, the proportion of patients with Hb level between 10 to 12 g/dl was similar in the 2 groups.

Consistent with previous reports, we observed that extended hours HD decreased serum phosphate levels, along with a reduced need for phosphate binders.^2^^,^^8^^,^^16^^,^^41^ Despite similar parathyroid hormone levels, both vitamin D and calcimimetic use decreased in the HHD group, reflecting better control of secondary hyperparathyroidism.

We found that HHD was associated with an increase in serum albumin level and postdialysis body weight. FHN Nocturnal Trial found no difference in nutritional parameters.^42^ However, a meta-analysis showed an increase in serum albumin and intakes of protein/energy with nocturnal HHD.^8^ Increased appetite with intensive HD can lead to higher salt intake^43^ and higher interdialytic weight gain, as observed in this study. Lower ultrafiltration rate in the HHD group due to longer sessions may be a contributing factor to better survival. Despite a possible increase in protein intake, higher serum bicarbonate level observed in this study displays the superiority of intensive HD regarding the control of acidosis.^44^

Earlier studies showed lower levels of interleukin-6 and high sensitivity CRP along with an improvement of erythropoiesis-stimulating agents response with nocturnal HHD.^45^^,^^46^ Higher uremic toxin/inflammatory substance clearance with extended HHD may be the responsible mechanism for less inflammation. In support of these findings, our results favored HHD regarding the change in neutrophil/lymphocyte ratios.

Our data showed better BP control with HHD, in accordance with previous studies.^1^^,^^2^^,^^6^^,^^16^ BP was well controlled in both groups, which is likely to be the result of some nationwide adoption of strict volume control policy applied at Ege University.^47^^,^^48^ Although data on antihypertensive drug use were available, we could not definitively determine whether diuretics, beta-blockers, angiotensin-converting-enzyme inhibitors, angiotensin receptor blocker drugs were prescribed for hypertension or to maintain residual diuresis, treat or prevent CVD. Therefore, this was not included in the analyses.

Admittedly, our study has some limitations. Considering that this is an observational study, a cause-effect relationship cannot be claimed. Despite the use of propensity score-matched design and comparable transplantation rates in the groups, we cannot exclude residual confounding and undetected differences between the groups (i.e., education, motivation, social support, employment, income, or mobility). Although studying in prevalent HD patients may lead to immortal time bias, we used ESKD duration as a matching variable to diminish this bias; in addition, the results were adjusted by ESKD time in multivariate analysis. Although we do not have data, it is likely that most of the population did not have significant residual kidney function, given that the patients included had a long duration of ESKD. We did not measure patients’ adherence to treatment, but noncompliance is unlikely because the payer in the country imposes heavy penalties if the frequency or duration of HD sessions is less than planned.

In conclusion, this study shows that compared to ICHD, extended HHD is associated with better patient survival. HHD may reduce hospitalization and medication requirement, along with better control of BP and phosphate, and improvement in nutrition and inflammation.

## Appendix

### List of members of The Turkish Home Hemodialysis Initiative

**Table:** 

Full Name	Highest Academic Degrees	Affiliation
Fatma Toz	Head Nurse	Fresenius Medical Care, Izmir, Turkey
Huseyin Toz	MD	Ege University, Izmir, Turkey
Mehmet Ozkahya	MD	Ege University, Izmir, Turkey
Meltem Sezis	MD	Ege University, Izmir, Turkey
Mumtaz Yilmaz	MD	Ege University, Izmir, Turkey
Mehmet Sukru Sever	MD	Istanbul University, Istanbul, Turkey
Alaattin Yıldız	MD	Istanbul University, Istanbul, Turkey
Sıddig Momin Adam	MD	Fresenius Medical Care, Adana, Turkey
Mine Besler	MD	Fresenius Medical Care, Istanbul, Turkey
Handan Ogunc	MD	Fresenius Medical Care, Istanbul, Turkey
Mujdat Batur Canoz	MD	Fresenius Medical Care, Yalova, Turkey
Mustafa Eren	MD	Fresenius Medical Care, Antalya, Turkey
Melih Anil	MD	Fresenius Medical Care, Ankara, Turkey
Kezban Pinar Yeniay	MD	Fresenius Medical Care, Bursa, Turkey
Ismail Ozer	MD	Fresenius Medical Care, Samsun, Turkey
Pınar Ergin	MD	Fresenius Medical Care, Adana, Turkey
Elif Arı Bakır	MD	Fresenius Medical Care, Istanbul, Turkey
Habib Emre	MD	Fresenius Medical Care, Balıkesir, Turkey
Hüseyin Atalay	MD	Fresenius Medical Care, Mersin,Turkey
Cemal Kurt	MD	Fresenius Medical Care, Mersin,Turkey
Fatma Adam	MD	Fresenius Medical Care, Adana, Turkey
Pinar Seymen	MD	Fresenius Medical Care, Istanbul, Turkey
Numan Görgülü	MD	Fresenius Medical Care, Istanbul, Turkey
Bahtisen Guven	MD	Fresenius Medical Care, Istanbul, Turkey
Mustafa Keleş	MD	Fresenius Medical Care, Antakya, Turkey

## Disclosure

EO is a medical/scientific consultant for Fresenius Medical Care (FMC) Turkey. CD, FK, SE, and EM are employees in FMC Turkey Clinics. SKK is the medical director of FMC Turkey. SS is an employee of FMC and holds stock in FMC. FWM is an employee of FMC, member of FMC, American Council on Germany, Vifor Fresenius Medical Care Renal Pharma, Humacyte (Board Observer); and holds stock in FMC. JGR is an employee of the Renal Research Institute, a wholly owned subsidiary of FMC, and owns shares of stock in FMC. PK is an employee of the Renal Research Institute, a wholly owned subsidiary of FMC; PK holds stock in FMC. PGK has honoraria from Astra-Zeneca and honorary Treasurer for the Australia New Zealand Society of Nephrology. CTC holds the R Fraser Elliott Chair in Home Dialysis and serves as consultant to Medtronic, Quanta and Dialco Inc; he received an investigator-initiated grant from Medtronic ERP program. GA, KY and ARO have declared no conflicting interest.

## Supplementary Material

Supplementary File (PDF)

**Table S1.** Variables before and after matching procedure in the treatment groups.

**Table S2.** HHD discontinuation reasons.

**Table S3.** Baseline characteristics of the patients who had been transplanted from 2 groups.

**Figure S1.** Flowchart, group 1.

**Figure S2.** Flow chart, group 2.

**Figure S3.** Flow chart, group 3.

**Figure S4.** Flow chart, group 4.

**STROBE Checklist.**
